# Renal Replacement Therapy in Pediatric Cardiac Intensive Care: A Retrospective Analysis of Modalities, Outcomes, and Prognostic Factors

**DOI:** 10.3390/jcm14207238

**Published:** 2025-10-14

**Authors:** Joanna Michalczuk, Sylwia Turek, Anna Jander, Marcin Tkaczyk

**Affiliations:** 1 Department of Pediatrics, Immunology and Nephrology, Polish Mother’s Memorial Hospital-Research Institute (ICZMP), 93-338 Łódź, Poland; joanna.michalczuk@iczmp.edu.pl (J.M.);; 2 Department of Pediatrics, Nephrology and Immunology, Medical University of Łódź, 93-338 Łódź, Poland

**Keywords:** acute kidney injury, pediatric cardiac surgery, dialysis, renal replacement therapy, ECMO

## Abstract

**Background**: Acute kidney injury (AKI) frequently complicates the postoperative course in pediatric patients after cardiac surgery and may necessitate renal replacement therapy (RRT). Despite the increasing use of RRT in this population, data on its modalities, outcomes, and prognostic factors remain limited. **Methods**: This retrospective cohort study included 37 children (aged 2 days–14 years) who underwent RRT in a cardiac intensive care unit (CICU) over a 35-month period. Modalities used were continuous veno-venous hemodiafiltration (CVVHDF) and peritoneal dialysis (PD). **Results**: The overall mortality was 76%, with no significant difference between RRT modalities. CVVHDF was used in 84% of cases, often during ECMO support. PD was more common in neonates and low-weight infants. Fluid overload and anuria were the leading indications. Survivors were older and heavier. Technical parameters, including blood flow, dialysis dose, and anticoagulation method, were not associated with survival. **Conclusions**: RRT in pediatric CICU patients is associated with high mortality, independent of modality. Early identification and appropriate patient selection may improve outcomes.

## 1. Introduction

Acute kidney injury (AKI) following cardiac surgery in children with congenital or acquired heart disease is a common complication in the intensive care unit (ICU), with an incidence reported between 40% and 60% of cases [1]. It affects children across all age groups but is particularly prevalent among neonates and young infants [2]. AKI is associated with increased mortality, prolonged hospitalization, and an elevated risk of developing chronic kidney disease (CKD) [3].

Risk factors for AKI can be classified into preoperative, intraoperative, and postoperative categories. These include age below 12 months, low body weight, reduced preoperative glomerular filtration rate (GFR), comorbidities such as diabetes and anemia, mechanical ventilation, prolonged renal ischemia, perioperative use of nephrotoxic drugs and vasopressors, pulmonary hypertension, cyanotic heart defects, use and duration of extracorporeal membrane oxygenation (ECMO), reoperation, prolonged aortic crossclamp time, and sepsis [3,4,5]. Reported mortality in children with AKI after cardiac surgery ranges from 20% to 79% [5,6,7,8].

The frequency of AKI requiring renal replacement therapy (RRT) after cardiac surgery varies between 1% and 17%, depending on the complexity of the procedure [5,9]. There are no universally accepted criteria for initiating RRT; decisions are made on a case-by-case basis by the clinical team, considering parameters such as fluid overload, anuria, electrolyte imbalance, and serum levels of urea and creatinine. Some studies suggest that starting RRT when fluid overload is less than 10% may improve survival [9]. Statistical analyses show that children who receive RRT typically present with stage 2 or 3 AKI, according to the KDIGO classification [7].

There are four main types of RRT modalities used in pediatric intensive care: intermittent hemodialysis (HD), and three continuous techniques—continuous veno-venous hemodialysis (CVVHD), continuous veno-venous hemofiltration (CVVH), and continuous veno-venous hemodiafiltration (CVVHDF). Peritoneal dialysis (PD) is also widely used. In cardiac ICUs, CVVHDF and PD are the most commonly implemented due to their availability and applicability in hemodynamically unstable patients. Although intravascular access methods are increasingly preferred in general pediatric ICUs, PD is still frequently chosen in children after cardiac surgery due to their younger age and smaller size [8,9].

CVVHDF is often the preferred modality because it allows gradual removal of fluids and solutes. In contrast to HD, which rapidly removes large volumes over a short period, CVVHDF enables controlled, continuous clearance that can be adjusted to the patient’s clinical status. It is especially useful in hemodynamically unstable patients with heart failure and allows for more flexible fluid management, including intravenous medications, parenteral nutrition, and blood product transfusions [1,10].

The efficacy of CRRT depends largely on the quality of vascular access, which is necessary for achieving adequate blood flow through the circuit [7]. Most commercially available CRRT devices are not licensed for use in children under 8 kg, making vascular access technically challenging or even unfeasible in such cases. In these situations, ECMO can be used as a bridge to support circulation and enable RRT. A dedicated device for neonates, the CARPEDIEM system (Medtronic), is now used in about 15% of high-specialization European centers [7].

One major advantage of PD is that it can be performed even in very small infants. Some institutions apply a prophylactic strategy by initiating PD postoperatively in high-risk groups [5]. PD is also the least costly of all dialysis methods and can be conducted manually without the need for an automated cycler [10]. It does not require anticoagulation or vascular access, making it suitable for patients with coagulopathies or difficult vascular access. However, compared to CVVHDF, PD is less efficient and slower in removing fluids and toxins, as its efficacy depends on the patient’s peritoneal membrane capacity [7]. Additionally, patients with limited lung capacity or respiratory disorders may not tolerate PD well due to increased diaphragmatic pressure. Children with diaphragmatic defects, prior abdominal surgeries, or peritonitis are also poor candidates for this modality [10].

## 2. Materials and Methods

This study was designed as a retrospective analysis of medical records of pediatric patients treated with renal replacement therapy (RRT) in the Cardiac Intensive Care Unit of a tertiary pediatric cardiac surgery referral center in Poland, over a 35-month period from November 2021 to September 2024.

Patient records were reviewed for details including the type of congenital defect and cardiac intervention performed, use of diuretics, need for mechanical ventilation, indications for dialysis, use of extracorporeal membrane oxygenation (ECMO), dialysis modality and type of vascular access, types of filters used, dialysis settings, anticoagulation methods, duration of therapy, complications, and outcomes including renal function recovery and overall survival.

Patient selection and study objectives. All consecutive pediatric patients (<18 years) who required RRT during hospitalization in the Cardiac Intensive Care Unit between November 2021 and September 2024 were included. No selection was performed using ICD-9/ICD-10 codes. Exclusion criteria were: preexisting chronic kidney disease (CKD) or end-stage renal disease, as the study focused exclusively on AKI occurring in the context of critical cardiac conditions. The primary objective of the study was to describe the use of RRT modalities, indications, and outcomes in critically ill pediatric cardiac patients. Secondary objectives were to evaluate prognostic factors associated with mortality and renal recovery.

AKI was defined according to the 2012 KDIGO criteria: an increase in serum creatinine by ≥0.3 mg/dL (26.5 µmol/L) within 48 h, or a 1.5-fold increase in serum creatinine within 7 days, or urine output <0.5 mL/kg/h for at least 6 h [1].

The decision to initiate RRT was made by a multidisciplinary team—including an intensivist, pediatric nephrologist, and pediatric cardiac surgeon—in the presence of severe oliguria/anuria, fluid overload unresponsive to diuretics, significant electrolyte disturbances (particularly hyperkalemia), or progressive metabolic acidosis.

Choice of RRT modality was guided by patient size, hemodynamic stability, and institutional practice. Continuous veno-venous hemodiafiltration (CVVHDF) was preferred in hemodynamically unstable patients and in those weighing > 5 kg. Peritoneal dialysis (PD) was most often used in neonates and small infants, particularly in the immediate postoperative period after cardiac surgery, or when vascular access for CRRT was not feasible. Intermittent hemodialysis (IHD) was not routinely performed due to patient instability and technical limitations.

RRT was discontinued when satisfactory diuresis (≥2 mL/kg/h) was achieved, kidney function parameters stabilized or improved, or when the patient deceased.

Continuous variables are presented as means with standard deviations (SD) or medians with interquartile ranges (IQR), as appropriate. Categorical Variables are presented as counts and percentages. Group comparisons were performed using the Mann–Whitney U test for continuous variables and Fisher’s exact test for categorical variables. Because of the limited sample size and small number of survivors, no multivariate analysis was performed, as it would have been statistically underpowered. Only descriptive and univariate comparisons were carried out.

The study was conducted at the Polish Mother’s Memorial Hospital-Research Institute (ICZMP) in Łódź, Poland. Ethical approval was waived by the institutional review board due to the retrospective and anonymized nature of the data.

Generative AI tools (such as ChatGPT version GPT-4 and GPT-5, developed by OpenAI) were used to assist in language editing and improving the clarity of expression in some parts of the manuscript. All content was reviewed and verified by the authors to ensure accuracy and integrity.

## 3. Results

### 3.1. Patient Characteristics

A total of 37 children (21 boys, 16 girls), aged from 2 days to 14 years (mean age: 12 months), and weighing between 2.7 and 38 kg (mean: 6.5 kg), rwent renal replacement therapy in the Cardiac Intensive Care Unit. All patients required RRT in the postoperative period following cardiac surgery. In 68% of cases, extracorporeal membrane oxygenation (ECMO) was used. Continuous veno-venous hemodiafiltration (CVVHDF) was applied in 84% (31 patients), while peritoneal dialysis (PD) was used in 16% (6 patients). One patient required both modalities during the same hospital stay. Selected clinical and biochemical data are presented in Table 1. We collected information on fluid intake during the 24 h prior to dialysis initiation and found no significant differences in this parameter. Overhydration, calculated as the relative increase in body weight between ICU admission and the day of RRT initiation, was available in 20 patients (mean: 14%; range: 8–24%). No significant differences were observed between survivors and non-survivors, or between patients treated with CRRT and those treated with PD.

Data are shown for the entire cohort (*n* = 37), the CVVHDF subgroup (*n* = 31), and the peritoneal dialysis subgroup (*n* = 6). Values are presented as mean and range. A statistically significant difference was observed between the groups for age, weight, body surface area, and duration of ECMO before dialysis (*p* < 0.05). Data are presented as mean (minimum–maximum) unless otherwise specified. Laboratory values are presented for the entire cohort and by dialysis modality. ALT levels were significantly higher in the CVVHDF group compared to PD (*p* < 0.05).

**Figure 1:**
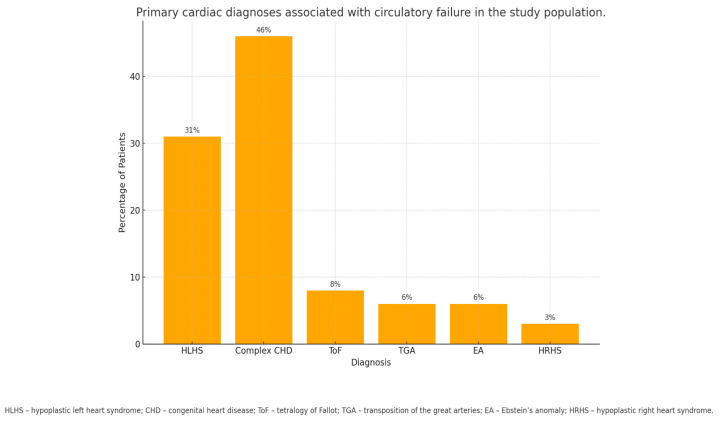
Primary cardiac diagnoses associated with circulatory failure in the study population.

Cardiac defects were the primary cause of ICU admission in 95% of cases. The most common was hypoplastic left heart syndrome (HLHS, 7 patients). Other congenital defects included tetralogy of Fallot (ToF), transposition of the great arteries (TGA), hypoplastic right heart syndrome (HRHS), and Ebstein’s anomaly (EA), comprising 46% of all diagnoses. The distribution of congenital heart defects in the study population is shown in Figure 1. Only two patients were hospitalized for non-cardiac reasons: one for post-ablation complications and another with congenital third-degree atrioventricular block, requiring pacemaker implantation during the first 24 h of life.

### 3.2. CVVHDF Group Outcomes

CVVHDF was applied in 31 patients (18 boys, 13 girls), with a mean age of 14.4 months (range: 2 days to 14 years) and a mean body weight of 7.12 kg. The average body surface area was 0.36 m^2^. Dialysis was performed using PrismaFlex or PrisMax (Baxter, Deerfield, IL, USA) systems with filters (HF20, ST60, ST100) selected according to patient size and access type. Circuits were primed with a 1:1 solution of saline and irradiated leukocyte-depleted red blood cells to reduce sudden pressure drops and hematocrit changes [11,12].

Vascular access was obtained via ECMO in 55%, via central venous catheters in 16%, and both methods in 29%. Most patients started dialysis shortly after ECMO initiation; in 50% of cases, within the first 24 h. Mean CVVHDF duration was 216.5 h (range: 4–954.5 h). Mean blood flow was 8 mL/kg/min; dialysate flow 54 mL/kg/h; replacement fluid 19 mL/kg/h; and ultrafiltration rate 5 mL/kg/h. Dialysis dose averaged 200 mL/kg/h (Table 2).

Average values and ranges for blood flow, dialysate and replacement solution flow, ultrafiltration rate, and total dialysis dose are presented.

The standard anticoagulation protocol with unfractionated heparin for the CRRT circuit consisted of a 10–20 IU/kg bolus followed by a continuous prefilter infusion of 5–10 IU/kg/h. In patients on ECMO support, unfractionated heparin was administered according to the ECMO protocol, with no additional anticoagulation used in the CRRT circuit (initial bolus 50–100 IU/kg, continuous infusion 10–20 IU/kg/min). In both settings, dosage adjustments were based on repeated assessment of activated clotting time (ACT), aiming to maintain values between 150–200 s. For citrate anticoagulation, we used Prismocitrate 18/0 solution with an initial dosage of 3.0 mmol/L of blood flow. No consistent differences in anticoagulation type or dosage were observed between survivors and non-survivors.

Primary indications were anuria (51.6%), fluid overload (32%), and severe azotemia (10%). Pharmacologic diuresis was attempted in 65% of patients. Filter lifespan averaged 44.9 h. Circuit clotting, high filter pressures, and inflow alarms were the most common complications. Interruptions due to surgical procedures or imaging often exceeded recirculation safety limits, requiring circuit replacement.

### 3.3. Peritoneal Dialysis Group Outcomes

PD was used in 6 infants (mean weight: 3.38 kg, mean BSA: 0.19 m^2^), four of whom had previously been on ECMO but were no longer receiving it at the time of PD initiation. Indications were anuria (5 patients) and fluid overload (1 patient). Manual exchanges were started immediately after Tenckhoff catheter placement in the operating room. Exchange volume averaged 42.5 mL (12.65 mL/kg); dwell time averaged 78 min (Table 3).

Data include mean and range for exchange volume, volume per kilogram, and dwell time.

Due to low exchange volumes, none of the patients qualified for automated cyclers. Complications included catheter leakage in one patient and outflow dysfunction in another, requiring treatment discontinuation. Mean PD duration was 147 h. Four patients died; two recovered renal function.

### 3.4. Comparison Between Survivors and Non-Survivors

A summary of clinical and biochemical differences between survivors and non-survivors is presented in Table 4. PD patients were significantly younger and smaller in size. No significant differences in initial renal function or urine output were noted between groups. Alanine aminotransferase (ALT) levels were significantly higher in the CVVHDF group. RRT duration did not differ significantly between modalities.

Significant differences were noted in age, weight, body surface area, leukocyte count, fluid therapy volume before dialysis, and hospitalization duration (*p* < 0.05). Hospital stay was calculated from hospital admission to discharge or death.

### 3.5. Clinical Outcomes and Prognostic Parameters

Mortality data are summarized in Table 1, while the duration of RRT and hospital stay are presented in Table 4. The timing of dialysis initiation after surgery differed between modalities: in patients treated with CRRT, RRT was initiated at a mean of 3.9 days postoperatively (range: 1–18 days), while in the PD group the mean interval was 8.1 days (range: 1–14 days; *p* = 0.010). There was no significant difference in the timing of dialysis initiation after surgery between survivors and non-survivors (median 4.5 days [IQR: 2–7] vs. 4.8 days [range: 1–18]; *p* = 0.931). In our analysis, hospital stay was calculated from the time of hospital admission until discharge or death, not from the initiation of RRT. In addition, the median length of CICU stay was 16 days (IQR: 12–33). Overall, 28 of 37 patients (75.7%) died during hospitalization. In long-term follow-up, all surviving patients had recovered normal renal function and none required chronic dialysis. Mortality did not differ significantly between dialysis modalities. Survivors were older, heavier, and had higher leukocyte counts. No significant differences were observed in dialysis parameters or duration of ECMO. Filter lifespan and dialysis settings did not impact survival. Technical parameters—such as blood flow, dialysis dose, and anticoagulation—were not significantly different between survivors and non-survivors. Filter lifespan was below 72 h in both groups. RRT duration had no significant impact on survival. Although older age appeared to be associated with better survival, this observation could not be confirmed in multivariate analysis, which was not feasible due to the small cohort size.

## 4. Discussion

In this cohort of 37 pediatric patients treated in a cardiac intensive care setting, the observed mortality associated with renal replacement therapy (RRT) was 75.7%, and it did not differ significantly depending on the dialysis modality used. Compared to the published literature, this is among the highest reported mortality rates. For example, Sanchez-de-Toledo et al. reported mortality ranging from 25% to 44.4%, with lower rates observed in patients undergoing peritoneal dialysis [9]. Santiago and colleagues noted a 43% mortality rate [8]. These discrepancies may be attributable to later RRT initiation in our center, or the more severe clinical profiles of our patients, who predominantly consisted of neonates and infants with complex cardiac anomalies such as hypoplastic left heart syndrome (HLHS).

Our findings should also be interpreted in the context of multicenter studies. Mortality in our cohort (75.7%) was higher than the 40–60% typically reported in larger pediatric series of critically ill children receiving RRT [13,14]. This discrepancy is likely related to the specific characteristics of our population, consisting exclusively of postoperative pediatric cardiac patients, many of whom required ECMO support. Importantly, the complete renal recovery observed in all long-term survivors in our study is consistent with previous multicenter reports, although follow-up beyond hospital discharge has not been systematically documented [15,16].

While some studies suggest that early initiation of RRT can improve outcomes, this approach also increases the cost of care and raises the risk of overtreating certain patients. Our findings support the observation that early initiation might be beneficial but must be carefully balanced against clinical risk.

In this analysis, low body weight, small body surface area, and lower leukocyte count were associated with higher mortality. Other studies have identified additional risk factors, including delayed initiation of RRT, hypotension, elevated creatinine at initiation, oliguria, sepsis, and multiorgan failure [4,7,13,17]. The lack of statistical significance for these variables in our dataset may be due to the small and relatively homogeneous nature of our patient population. Nevertheless, some of the key risk factors identified in the literature were confirmed in our study.

CVVHDF was the most commonly used modality (68%), largely because many patients were already connected to ECMO, which provided vascular access for extracorporeal dialysis. In contrast, Sanchez-de-Toledo et al. reported a 78% rate of PD use in similar patients [9]. Guzzo et al. also observed that continuous modalities are now the standard in pediatric ICUs, while PD is still commonly used after cardiac surgery [15].

In our center, peritoneal dialysis was initiated only after clinical signs of kidney failure appeared. Other centers implement prophylactic catheter placement during surgery, which may or may not be followed by dialysis, depending on fluid balance. This strategy has been associated with lower mortality in some single-center studies, especially in neonates [18], though multicenter data do not always confirm these benefits [15,16]. Ongoing efforts aim to establish clinical criteria for qualification [16,18]. According to the authors, such criteria support appropriate patient selection for prophylactic peritoneal dialysis catheter placement, which may have important practical implications.

From a technical perspective, our PD approach relied on manual exchanges with small volumes and frequent cycles. This is consistent with protocols described by Namachivayam and Kwiatkowski et al. [17,18]. Such treatment requires specialized supplies that are often available only in high-level clinical centers. Our average dwell time (78 min) was slightly longer than in other studies [17,18], but this did not appear to affect biochemical or clinical efficacy. The absence of rigid international standards allows for some flexibility in procedure planning.

CVVHDF was our preferred continuous method, although no study has demonstrated a clear advantage among CRRT subtypes (CVVHD, CVVH, CVVHDF) [19]. Thus, the choice is typically guided by clinical experience and institutional resources. Our dialysis settings were consistent with published recommendations. For example, our mean blood flow rate (around 8 mL/kg/min) aligns with findings by Askenazi et al. [20].

Most patients in our study were on ECMO, which necessitated systemic anticoagulation—typically unfractionated heparin—a practice also described by Sanchez-de-Toledo and Pistolesi et al. [9,19]. Filter lifespan was generally shorter than the recommended 72 h, which is also consistent with other pediatric CRRT studies [14,15,20].

It should be noted that our center did not have access to the CARPEDIEM device during the study period. Designed specifically for neonates over 2.5 kg, this system is not yet widely adopted. Whether it would improve outcomes in this high-risk group remains an open question, as evidence from clinical trials is still lacking.

Limitations: This study has several limitations. First, its retrospective and single-center design inherently limits the generalizability of the findings, and the results should be interpreted with caution. Second, standardized severity of illness scores such as PELOD or PRISM were not routinely collected in our unit and therefore could not be analyzed. This lack of standardized illness severity assessment may have influenced the evaluation of baseline clinical status and the interpretation of outcomes. Finally, fluid balance data before and during RRT were not systematically available, preventing us from assessing its prognostic impact. Moreover, due to the limited cohort size, our study was underpowered to perform multivariate analysis, and potential confounders such as age could not be formally adjusted. Larger, prospective multicenter studies with systematic collection of severity scores and fluid balance are needed to confirm and extend our findings.

## 5. Conclusions

This retrospective analysis of renal replacement therapy (RRT) in a tertiary pediatric cardiac surgery referral center clearly demonstrates that the primary indications for initiating RRT in children after cardiac surgery are fluid overload and anuria. The experience and technical capabilities of the multidisciplinary care team enable the effective implementation of both peritoneal dialysis and continuous renal replacement therapies in neonates, infants, and children.

Peritoneal dialysis tends to be preferred in younger, lower-weight patients, especially when ECMO support is not required. However, the choice of dialysis modality did not significantly impact survival in this cohort.

Renal replacement therapy in pediatric cardiac intensive care units is complex and associated with a high risk of clinical failure. The mortality rate in this patient population remains high (77%) and appears to be independent of the dialysis method used. Importantly, RRT was always initiated after the onset of clinical indications, and prophylactic initiation was not practiced in our cohort.

## Figures and Tables

**Table 1:** Demographic, clinical and biochemical characteristics of the study cohort at dialysis initiation.

Feature	Total (*n* = 37)	CVVHDF (*n* = 31)	PD (*n* = 6)	*p* Value
**Sex (M:F)**	21:16	18:13	3:3	0.128
**Age (months), mean (min–max)**	12.36 (0–168)	14.4 (0–168) *	1.58 (0.5–4)	0.023
**Weight (kg), mean (min–max)**	6.5 (2.7–38)	7.12 (2.85–38) *	3.38 (2.7–4.5)	0.004
**BSA (m^2^), mean (min–max)**	0.32 (0.16–1.46)	0.36 (0.17–1.46) *	0.19 (0.16–0.27)	0.003
**Daily urine output (ml), mean (min–max)**	241 (0–1120)	254 (0–1120)	181 (35–345)	0.197
**ECMO before dialysis (days), mean (min–max)**	4.81 (1–18)	3.86 (1–18) *	10 (6–14)	0.010
**ECMO during dialysis (%)**	40.5%	80.64%	0%	0.001
**Urea (mg/dL)**	98.5 (25–284)	98.4 (25–284)	98.6 (62–126)	0.450
**eGFR (mL/min/1.73 m^2^)**	31.25 (0–86)	31.9 (0–86)	27.5 (0–46.7)	0.271
**ALT (IU/L)**	295.06 (5–2169)	340.54 (5–2169) *	82.8 (8–420)	0.031
**Mortality (%)**	75.7	77.4	66.6	0.607

Abbreviations: CVVHDF—continuous veno-venous hemodiafiltration; PD—peritoneal dialysis; ECMO—extracorporeal membrane oxygenation; BSA—body surface area; ns—not significant; eGFR—estimated glomerular filtration rate; ALT—alanine aminotransferase. * *p* < 0.05.

**Table 2:** CRRT technical parameters in patients treated with CVVHDF (*n* = 31).

Parameter	Mean	Min	Max
**Blood flow (mL/min)**	42.90	10	100
**Blood flow/kg (mL/kg/min)**	7.86	1.85	14.28
**Dialysate flow/kg (mL/kg/h)**	53.95	9.25	131.57
**Replacement flow/kg (mL/kg/h)**	19.13	5.4	34.48
**Ultrafiltration/kg (mL/kg/h)**	5.32	1.06	10.6
**Dialysis dose (mL/kg/h)**	78.40	20.37	150.0

Abbreviations: CVVHDF—continuous veno-venous hemodiafiltration.

**Table 3:** Peritoneal dialysis parameters in the PD group (*n* = 6).

Parameter	Mean	Min	Max
**Exchange volume (mL)**	42.5	30	60
**Volume/kg (mL/kg)**	12.68	8.82	15
**Dwell time (min)**	78	60	120

Abbreviations: PD—peritoneal dialysis.

**Table 4:** Clinical and biochemical comparisons between survivors and non-survivors.

Parameter	Deaths	Survivors	*p* Value
**Age (months)**	4.4 (0–39) *	37.2 (0–168)	0.005
**Weight (kg)**	4.7 (2.7–11) *	12.1 (3–38)	0.003
**BSA (m^2^)**	0.3 (0.16–0.53) *	0.5 (0.16–1.46)	0.003
**ECMO before dialysis (days)**	4.8 (1–18)	4.5 (2–7)	0.432
**Dialysis duration (h)**	223.0 (4–954.5)	150.4 (59–372)	0.332
**Filter lifespan (CRRT, h)**	41.8 (4–72)	55.7 (28.38–77)	0.081
**Dialysis dose (ml/kg/h)—CRRT only**	86.2 (20.3–150.0)	62.2 (46.6–85.3)	0.211
**WBC (10^3^/µL)**	10.9 (1.93–37.73) *	18.5 (11.53–41)	0.045
**ALT (IU/L)**	241.5 (8–2169)	469.1 (5–1730)	0.504
**Fluid therapy before dialysis (mL)**	663.6 (159–1915) *	1526.4 (315–3001)	0.001
**Fluid therapy (mL/kg/24 h)**	151.0 (29.44–284.28)	152.9 (56.84–321.66)	0.641
**Hospital stay (days)**	39.6 (5–109) *	61.9 (31–119)	0.043

Abbreviations: BSA—body surface area; ECMO—extracorporeal membrane oxygenation; CRRT—continuous renal replacement therapy; WBC—white blood cell count; ALT—alanine aminotransferase. * *p* < 0.05.

## Data Availability

The data presented in this study are not publicly available but are available on reasonable request from the corresponding author.

## Abbreviations

The following abbreviations are used in this manuscript:

AKI	Acute Kidney Injury;
ALT	Alanine Aminotransferase;
BSA	Body Surface Area;
CKD	Chronic Kidney Disease;
CICU	Cardiac Intensive Care Unit;
CVVHDF	Continuous Veno-Venous Hemodiafiltration;
ECMO	Extracorporeal Membrane Oxygenation;
EA	Ebstein’s Anomaly;
HF	Hemofilter;
HLHS	Hypoplastic Left Heart Syndrome;
HRHS	Hypoplastic Right Heart Syndrome;
ICU	Intensive Care Unit;
ICZMP	Polish Mother’s Memorial Hospital-Research Institute;
KDIGO	Kidney Disease: Improving Global Outcomes;
PD	Peritoneal Dialysis;
RRT	Renal Replacement Therapy;
TGA	Transposition of the Great Arteries;
ToF	Tetralogy of Fallot.
